# Pacific biosciences sequencing technology for genotyping and variation discovery in human data

**DOI:** 10.1186/1471-2164-13-375

**Published:** 2012-08-05

**Authors:** Mauricio O Carneiro, Carsten Russ, Michael G Ross, Stacey B Gabriel, Chad Nusbaum, Mark A DePristo

**Affiliations:** 1 Broad Institute of MIT and Harvard, Medical and Population Genetics Program, 301 Binney St, Cambridge, MA, 02141, USA; 2 Broad Institute of MIT and Harvard, Genome Sequencing and Analysis Program, 320 Charles St, Cambridge, MA, 02141, USA

## Abstract

**Background:**

Pacific Biosciences technology provides a fundamentally new data type that provides the potential to overcome some limitations of current next generation sequencing platforms by providing significantly longer reads, single molecule sequencing, low composition bias and an error profile that is orthogonal to other platforms. With these potential advantages in mind, we here evaluate the utility of the Pacific Biosciences RS platform for human medical amplicon resequencing projects.

**Results:**

We evaluated the Pacific Biosciences technology for SNP discovery in medical resequencing projects using the Genome Analysis Toolkit, observing high sensitivity and specificity for calling differences in amplicons containing known true or false SNPs. We assessed data quality: most errors were indels (~14%) with few apparent miscalls (~1%). In this work, we define a custom data processing pipeline for Pacific Biosciences data for human data analysis.

**Conclusion:**

Critically, the error properties were largely free of the context-specific effects that affect other sequencing technologies. These data show excellent utility for follow-up validation and extension studies in human data and medical genetics projects, but can be extended to other organisms with a reference genome.

## Background

Massively parallel DNA sequencing technologies power many of today’s studies of genetic traits, including population diversity projects like the 1000 Genomes (1000 G) [1], the sequencing-based successors to disease association studies [2-5], and mutation discovery in tumors (reviewed in [6-9]). The new data types they provide are far cheaper than traditional capillary gel electrophoresis data, with the reduction in cost enabling projects of unprecedented scale. However, current sequencing technologies such as Illumina, 454, SOLiD and Ion Torrent have systematic shortcomings that limit utility of the data. Each of these has its own complex context-specific error profile that results in false positive SNP and indel calls. Short read lengths can result in mismappings and misalignments [10,11] that make some regions of the genome inaccessible. In addition, there are representational biases, where regions of certain base compositions (in particular very high or very low GC composition) are underrepresented.

The Pacific Biosciences technology provides a fundamentally new data type that provides the potential to overcome these limitations by providing significantly longer reads (now averaging >1 kb), enabling more unique seeds for reference alignment. In addition, the lack of amplification in the library construction step avoids a common source of base composition bias. With these potential advantages in mind, we here evaluate the utility of the Pacific Biosciences RS platform for human medical amplicon resequencing projects by assessing the quality of the raw sequencing data, as well as its use for SNP discovery and genotyping using the Genome Analysis Toolkit (GATK)[10].

## Results and discussion

**Figure 1:**
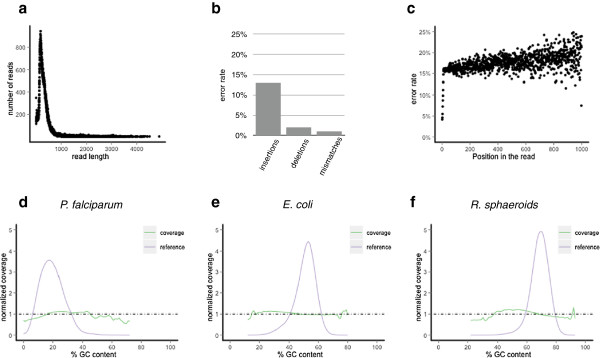
**Characterization of Pacific Biosciences data.a**) Base error mode rate for deletions, insertions and mismatches. **b**) Length distribution of reads in the Pacific Biosciences discovery dataset (here some raw reads are as long as 5,000 bases). **c**) Pacific Biosciences error rate by position. Shown are all errors (mismatch, insertion and deletion) by base position, including every base sequenced despite any previously known variation (this is why the average is slightly higher than 15%). Due to the diminishing number of reads with bases beyond 1000 we only plot here positions up to 1000. **d**-**f**) GC bias of the Pacific Biosciences instrument represented by the genomes of *P. falciparum* (low GC), *E. coli* (average GC) and *R. sphaeroides* (high GC) shows good balance in GC coverage where there is sufficient data in the genome, regardless of GC content.

We first defined the basic performance parameters of the data, in terms of read length, base accuracy and error profile, and sequence composition-based representational bias. Using a dataset of 4 runs of data (47,638 reads) from human amplicons (see methods), we profiled read length, showing an average of 700 bases over a wide distribution, including 5% of reads >2500 bases (Figure 1a). Errors were characterized by comparison to the known reference sequences (see methods), showing that the primary error mode was insertions, at 12%, followed by deletions, 2% and apparent mismatch errors at 1% (Figure 1b). Further, errors were randomly distributed across the read, rather than increasing in distal positions as is true for other data types (Figure 1c). This property by which read length and base position do not affect the quality of the bases produced greatly facilitates alignment of reads despite their relatively high indel rate. Finally, since extreme base composition is a source of representational bias in many sequence data types, we evaluated the performance of the Pacific Biosciences system using a dataset (12 runs, 319,090 reads) derived from 3 microbial genomes that span a much wider range of base composition than the human genome (*Plasmodium falciparum* 19% GC, *Escherichia coli* 50% GC, *Rhodobacter sphaeroides* 69% GC). The average relative coverage across GC content levels fell into ranges narrow enough to have little impact on data utility: between 53% and 110% of mean coverage for *P. falciparum*, between 79% and 140% for *E. coli*, and between 64% and 120% for *R. sphaeroides* (Figure 1d).

Base quality scores are a critical tool for accurate SNP calling, and are used by most analysis algorithms to help distinguish true variation from artifacts. Indeed, the accuracy of the reported base quality scores has a significant impact on the correctness of variation detection[12]. We have found that Pacific Biosciences data underreport true base qualities by on average 10 PHRED scaled points. For this reason we applied a method to generate empirical base quality scores for Pacific Biosciences data using the base quality score recalibration framework in the Genome Analysis Toolkit (GATK)[10]. Empirical quality scores generated with GATK are on average Q20-Q24 for bases that are not insertions or deletions. We observed no relationship between the empirical quality scores and position in the read, similar to accuracy of base calls.

**Figure 2:**
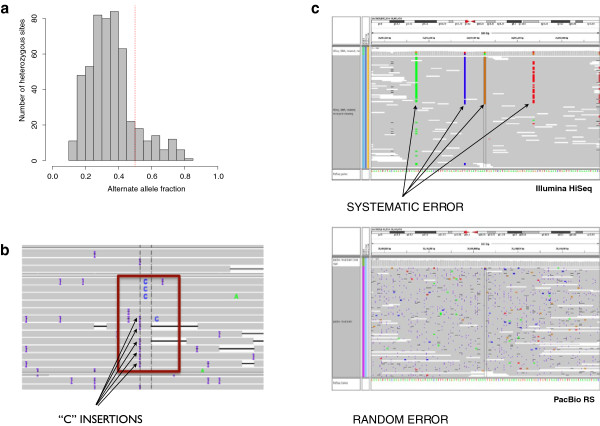
**Error profile of Pacific Biosciences data.a**) A chart showing the number of observations of the alternate allele in all heterozygous sites and how reference bias pulls the median significantly below the expected 0.5. This combination creates multiple possible alignments with the highest alignment score, allowing the aligner in some cases to hide the true alternate allele inside an insertion to maximize the alignment score at the cost of reference bias. **b**) IGV browser (http://www.broadinstitute.org/igv/) screenshot of the validation dataset showing an example of a case of aligner-created reference bias on Pacific Biosciences RS data. The true SNPs (C) are correctly called in individual reads. **c**) An IGV browser[18,19] screen snapshot of a region in the discovery dataset where Illumina HiSeq data suffers from context specific errors that makes it appear as a true heterozygous site whereas Pacific Biosciences RS data (with errors nearly random, though more frequent) clearly shows no event in this region.

Alignment is the process of mapping reads individually to a region in the reference sequence where each read can align with minimum number of mismatches and gap openings (insertions and deletions). Because of the high base insertion and deletion rates, alignment of Pacific Biosciences data challenges standard approaches. To account for this, we set the gap open penalty to be lower than the base mismatch penalty (see methods) in order to maximize alignment performance. Despite aligning most of the reads successfully, this creates the side effect that the aligner will sometimes prefer to “hide” a true SNP inside an insertion. The result is that mapping is accurate, but the aligned sequence is biased toward the reference at some indel positions (see Figure 2a and 2b), although the “hidden” bases remain available for subsequent analysis. It is important to note however, that reference bias is an artifact of the alignment process, rather than of the data, and can be greatly reduced by locally realigning the reads based on the reference and the data. Presently, the available software for local realignment is not compatible with the length and the high indel rate of Pacific Biosciences data, but we anticipate the development of new tools.

For all projects aimed at discovery of mutations or variation, it is critical to follow-up with validation of the variants called, either by sampling to determine the rate at which calls are correct, or to confirm which exact changes are real rather than art factual. Extension is an approach similar to validation, but carried out as a discovery process on samples that have not been sequenced to determine the extent to which mutations are present at the same base or in the same gene in a large number of samples, rapidly and at relatively low cost. Today, variants called by massively parallel sequencing are commonly validated using either Sequenom genotyping, Sanger sequencing or resequencing using an alternate massively parallel technology (e.g. variants called from Illumina HiSeq data are validated with 454 sequencing); for examples see[1,13-15]. Each of these options has limitations. Sequenom genotyping can validate only the specific mutation called, and so lacks the ability to identify base differences at sites that were not discovered (i.e. unable to validate mutation load assays, gene burden tests), and is thus much less effective for extension. Further, accurate interpretation of Sequenom data requires manual review, with the potential to introduce human error. Sanger sequencing operates at low throughput and also requires human interpretation of the sequencing results, making it only suitable for small validation assays. Using alternate sequencing platforms may not provide the fast turnaround necessary (Illumina HiSeq) for validation studies or may suffer from other context specific error modes (SOLiD, 454) that often further complicate the validation analysis. Sequencing with an alternate technology is, nevertheless, the most statistically correct approach for validation, provided the alternate sequencing methodology employs a different approach from the original technology, with a different spectrum of biases, error modes or other limitations.

We designed two targeted sequencing experiments to evaluate the power of the Pacific Biosciences data type as a tool for validation and extension and as a tool for variant discovery. For both experiments we used the GATK to discover sites (see methods for details on site selection).

To evaluate Pacific Biosciences data for validation/extension, we sequenced PCR-amplicons spanning 98 variant calls based on Illumina GA1 and GA2 data from the 1000 G project. These had been previously validated either as true *de novo* mutations (38 sites) or false call artifacts (60 sites), using SOLiD, 454 and Sanger sequencing chemistry[13]. Individual PCR amplicons spanning the test set variants were generated, sequenced with both the Pacific Biosciences RS and the Illumina MiSeq, and the data were used to call SNPs. Performance of data from the two platforms was similar by several metrics. Pacific Biosciences data showed 97% sensitivity and 98% specificity by correctly genotyping 96 of the 98 sites, with a negative predictive value of 98% and positive predictive value of 97%. Illumina MiSeq data provided 100% sensitivity and 91% specificity, genotyping 93 out of 98 sites correctly with 100% negative predictive value and 88% positive predictive value (see Tables 1 and 2). The miscalled sites from each data set were then manually inspected. One site was wrongly called monomorphic with the reference allele from Pacific Biosciences data due to reference bias. The one site wrongly called polymorphic from Pacific Biosciences data was similarly miscalled in MiSeq and in our gold standard HiSeq whole genome dataset (see methods). From the 5 sites miscalled using data from Illumina MiSeq, 2 were in agreement with Pacific Biosciences (one listed above and one not called in Pacific Biosciences due to reference bias), and 3 sites were called polymorphic in error due to noise in the MiSeq data (See Figure 2c for an example). Pacific Biosciences RS data performed well by all metrics, and at a similar quality to Illumina data, demonstrating that the RS is a powerful tool for follow up validation or extension.

**Table 1:** Validation calls for Pacific Biosciences and Illumina MiSeq

	**Pacific Biosciences RS**	**Pacific Biosciences RS**	**Illumina MiSeq**	**Illumina MiSeq**
	**called Poly**	**called Mono**	**called Poly**	**called Mono**
**Confirmed**	37	1	38	0
** *de novo* SNP**				
**Confirmed**	1	59	5	55
**artifact**				

Number of sites called polymorphic and monomorphic by Pacific Biosciences RS and Illumina MiSeq in the validation experiment. Datasets were sequenced from the same amplicons and were downsampled to 70x average coverage for comparison. Pacific Biosciences shows good accuracy with consistently high percentages in all metrics and making only 2 out 98 wrong calls, while MiSeq shows excellent sensitivity and negative predictive value but lower specificity and positive predictive value and 5 out of 98 wrong calls, all of the same class.

*Poly* = polymorphic site; *Mono* = monomorphic site.

**Table 2:** Validation metrics for Pacific Biosciences and Illumina MiSeq

	**Sensitivity**	**Specificity**	**PPV**	**NPV**
**Pacific Bioscinces RS**	97%	98%	97%	98%
**Illumina MiSeq**	100%	91%	88%	100%

*PPV* = positive predictive value; *NPV* = negative predictive value.

To evaluate Pacific Biosciences data for variant discovery, we sequenced 177 kb in 61 amplicons from regions across human chromosome 20 using both Pacific Biosciences RS and Illumina MiSeq. These amplicons contained 225 SNPs that had been validated in our gold standard deep whole genome call set (see methods), which includes 43 previously validated as high-confidence SNPs from Hap Map 3.3. A single Hap Map site was called with very high confidence by amplicon-based data from both technologies to have a different alternate allele than Hap Map, and was similarly called with the whole genome shotgun dataset. Thus, it likely reflects either a single base change in our DNA sample source or an error in Hap Map rather than a miscalled SNP. With Pacific Biosciences data we called 197 of the 225 gold standard sites (including 38 of the 43 Hap Map sites); with MiSeq data we called 222/225 (and 43/43). We manually inspected the sites of all the discordant calls, which fell into two classes: 1) *Reference bias*, where the correct call was obscured by reference bias in the alignment, and the alternate allele was present in the data but hidden by the alignment inside insertions; 2) *Low sequence coverage*, where insufficient data were obtained to make a confident call, likely due to poor PCR amplification of the target. For the Pacific Biosciences data, the 28 sites missed from the gold standard call set included 16 missing due to reference bias and 12 due to lack of coverage. For MiSeq data, the three sites missed were all due to low sequence coverage.

## Conclusions

Overall, this first look at Pacific Biosciences RS sequencing data from human samples demonstrates that it is a promising platform for follow-up validation and extension studies. We have shown that the data type is largely free from context-specific error modes and is effective both for site-specific validation and variation discovery. Despite its current error rate, the stochastic nature of the Pacific Biosciences error profile allows the standard Bayesian variant calling algorithm used by the GATK to make robust calls without any technology-specific modifications. Though Pacific Biosciences data are more expensive on a per base or per read basis than for MiSeq, for the experiments described herein, reagent costs are similar. We anticipate that future improvements in yield in the Pacific Biosciences technology and to the data processing tools in the GATK may overcome some of the remaining analytic challenges we found using Pacific Biosciences data which will further increase its utility in the field of human DNA sequencing.

## Methods

### Sample preparation

In this study we generated two datasets from the same individual (NA12878) from the 1000 G project, one for SNP validation and one for SNP discovery. For the validation dataset we sequenced 2,000 bp PCR amplicons containing 120 *de novo* mutation calls that had been identified using data from Illumina, SOLiD, and 454 sequencing technologies and subjected calls to follow-up validation. 48 sites were confirmed as true *de novo* mutations and 72 confirmed as false calls that in some cases required more than one round of validation. For the discovery dataset we sequenced 1,500 bp amplicons covering 61 regions of chromosome 20. The same amplicons were sequenced using Pacific Biosciences RS and Illumina MiSeq and down sampled to an average 70x coverage for direct comparison.

### Sequence data processing

We used the Pacific Biosciences SMART analysis software 1.2 to generate ‘filtered sub-reads’ from the instrument. The term ‘sub-read’ refers to the portion of a read from a single pass of the template. Filtering refers to a process within the software to identify quality reads. Filtered sub-reads are generated following primary analysis where the SMART bell adaptors are separated from the long raw reads, and low quality bases reported by the instrument are removed, yielding sub-reads 700 bases long on average. In the text we simply call sub-reads ‘reads’ to avoid confusion, and never refer to raw reads. We did not use the secondary analysis provided by the Pacific Biosciences SMART analysis software. Instead we used a BWA + GATK based pipeline to create analysis-ready BAM files from the original FAST file.

The pipeline starts with BWA-SW[16] read alignment using non standard parameters (−b5 -q2 -r1 -z20). These parameters were chosen after evaluating several other options for the best yield (number of reads), coverage of the targeted region, average read length and SNP-calling sensitivity (compared to standard calls made with Broad’s internal 60x coverage NA12878 HiSeq data). Read group information is then added to the SAM file and a BAM file is generated, sorted by coordinate order using Picard’s tools AddOrReplaceReadGroups and Sorts am respectively (picard.sourceforge.net). All datasets used in this work are available at: http://www.broadinstitute.org/gsa/wiki/index.php/The_Genome_Analysis_Toolkit.

### Quality score generation

Quality scores for the bases were generated by using the GATK’s base quality score recalibration pipeline[10], starting with a default original base quality of Q20 for every base in the read. Base quality score recalibration distributes the quality score according to the empirical quality score of each base. The empirical quality score is the likelihood that the base is correctly called based on four covariates: Cycle, Di-nucleotide, Base Quality and Read Group. The cycle covariate corrects the qualities based on the position of the base in the read. The Di-nucleotide covariate looks for systematic errors caused by a specific previous base call, for example, if calling a G usually makes the instrument more likely to call a subsequent C even if that is incorrect. The base quality covariate corrects for systematic variation in the quality of bases and the read group covariate rectifies inconsistencies in qualities between different sequencing runs. We tested this using the Pacific Biosciences SMART software (version 1.2) secondary analysis quality scores as original qualities for base quality score recalibration but observed no improvement in the final empirical quality scores. This effect is perhaps due to the fact that Pacific Biosciences does not provide a simple measure of base quality score. Instead the software uses the instrument’s estimated insertion error probability as the base’s quality score. We found that this value was often 5–10 PHRED-scaled points below the empirical quality score. We did not use the Pacific Biosciences reported base quality scores because their systematic underestimation of base qualities makes them less effective for variation detection.

### Variant calling

SNP calling was performed using the GATK’s Unified Genotyper with two added parameters to deal with the error mode and error rate of Pacific Biosciences reads. Since insertions and deletions are the primary error mode of Pacific Biosciences, we allowed sites to have up to 50% deletions with the parameter -deletions 0.8 and because the average quality scores for bases present ranged from 20–24 we allowed the minimum base quality score to be Q10 (PHRED-scaled) with the parameter -mbq 10. Due to the high coverage and small size of the datasets we did not observe any improvements in the calls by using hard (GATK’s Variant Filtration) or soft (GATK’s Variant Quality Score Recalibration pipeline) filters.

Validation was performed using GATK’s ‘Genotype And Validate’ tool using the Pacific Biosciences BAM files as input and a VCF file with the true call status of the variants in the region. We generated this VCF file using the GATK’s Variant Annotator and information from the previous validation experiments with Illumina, SOLiD and 454 data as part of the 1000 G project. Genotype And Validate makes calls using the GATK’s Unified Genotyper engine on every site in the validation VCF given the alternate allele in the VCF file and calculates the likelihood of calling this alternate allele using the Pacific Biosciences BAM file as input. The result is then compared with the true status of the alternate allele (true *de novo* or sequencing artifact). For each true *de novo* called with Pacific Biosciences data the tool outputs a true positive, for each sequencing artifact not called with Pacific Biosciences data the tool outputs a true negative. False positives are sequencing artifacts called with Pacific Biosciences and false negatives are true *de novo* events not called with Pacific Biosciences data.

Discovery was performed using the GATK’s Unified Genotyper directly with the parameters described above and Variant Eval to analyze the metrics of the call set (TiTv ratios, novel mutations, known mutations and other metrics). We used NA12878 genotypes in Hap Map 3.3 and our gold standard call set as our known datasets. The datasets, the GATK and all tools used in this work are freely available at http://www.broadinstitute.org/gsa/wiki/index.php/The_Genome_Analysis_Toolkit.

### Gold standard call set

The gold standard call set provided a highly reliable set of known control variants. It was produced from whole genome data sequenced to 60x from NA12878, and processed using the current best practices for variant calling as defined by the Broad Institute. The dataset was aligned using BWA 0.5.9-r16 and the 1000 G project b37 human reference. We realigned the reads using the GATK’s Target Creator and Indel Realigner, marked duplicate reads using Picard’s Mark Duplicates tool and recalibrated quality scores using the GATK’s Base Quality Score Recalibration pipeline consisting of the Count Covariates tool and the Table Recalibration tool. Calls were made using the GATK’s Unified Genotyper and filtered using the GATK’s Variant Quality Score Recalibration tool. The sequence data set and gold standard call set are available at http://www.broadinstitute.org/gsa/wiki/index.php/The_Genome_Analysis_Toolkit.

### Lab methods

PCR amplicons were generated as follows. PCR reactions were performed in a volume of 30 ul containing 30 ng DNA (NA12878, Coriell), 0.05 U PfuUltra II Fusion HS DNA polymerase (Agilent), 1x PfuUltra II Fusion buffer (Agilent), 0.4 uM primers (Integrated DNA Technologies), 0.66 mM dNTP (Agilent), 1% DMSO (VWR) and 1.15 M Betaine (Sigma-Aldrich). Using a thermal cycler (Master cycler ep gradient, Eppendorf) PCR was performed using 1 cycle of 95°C for 3 min, 40 cycles of 94°C for 30 sec, 55°C for 30 sec, and 72°C for 2 min followed by 1 cycle of 72°C for 10 min. PCR amplicons were quantified using a Quant-iT DNA Assay Kit, High Sensitivity (Invitrogen), normalized to the same concentration and 100 ng of each PCR product was combined into a pool. Pacific Biosciences sequencing libraries were generated from pooled PCR amplicons and sequenced on Pacific Biosciences RS following manufacturer’s recommendations. The 2000 bp amplicons were sheared to ~250 bp in size prior to library construction following manufacturer’s recommendations (Pacific Biosciences). The 1,500 bp amplicons were processed without shearing. Illumina paired-end fragment libraries were generated and sequenced on MiSeq following manufacturer’s recommendations (Illumina). Both 1,500 bp and 2,000 bp amplicons were sheared to ~200 bp in size following manufacturer’s recommendations (Illumina). For each amplicon pool library construction and sequencing were carried out independently. Data for *E. coliR. sphaeroides* and *P. falciparum* was generated on a previous study [17] where the lab methods are thoroughly described.

## Competing interests

The authors declare that they have no competing interests.

## Authors’ contributions

MC conceived and designed the study, performed the statistical analysis, developed the software, drafted the manuscript CR performed the molecular work and DNA sequencing MR performed the statistical analysis SG coordinated and participated in the design of the study CN designed the study and helped draft the manuscript MD conceived and designed the study and developed the software All authors read and approved the final manuscript.
